# Evaluation of Matrix Systems on the Proximal Contact of Class II Composite Restorations: A Systematic Review

**DOI:** 10.7759/cureus.50835

**Published:** 2023-12-20

**Authors:** Renad Alshardan, Amani Rozi, Dana AlSenan, Aseel Rozi, Buthaynah AlJohani, Jana Almusallam, Njoud AlAteeq

**Affiliations:** 1 Dentistry, Princess Nourah Bint Abdulrahman University, Riyadh, SAU; 2 Dentistry, Riyadh Elm University, Riyadh, SAU

**Keywords:** proximal tightness, class ii restoration, resin composite, proximal contact, matrix system

## Abstract

When restoring proximal surfaces in posterior teeth, dentists frequently encounter the difficulty of reproducing the proximal contact that is naturally present in unrestored teeth. In order to guarantee the durability of restorations made from class II composite resin, it is imperative that both functional and aesthetic requirements are met. This entails the choice of the matrix system that replicates the optimal proximal contact subsequent to the insertion of restorations made of class II composite resin. The objective of this systematic review is to assemble current research conducted on the various matrix systems utilized in class II composite restorations and assess their impact on the pre-existing composite proximal contact. Three examiners conducted an independent electronic search utilizing the subsequent databases: Web of Science, Cochrane Library, Scopus, PubMed, and Embase. Publications on proximal contact in class II composite restorations were compiled from the time of their inception until August 2022, in accordance with predetermined inclusion and exclusion criteria. The methodological quality assessment was conducted utilizing the Effective Public Health Practice Project (EPHPP) instrument. Among the six studies that were included, it was observed that the sectional matrix system generated greater proximal contact tightness than the circumferential matrix system (Tofflemire). However, no significant distinction was found between the metal and polyester matrix systems. In contrast to alternative circumferential matrix systems, the utilization of a sectional matrix system yields a statistically significant improvement in the optimum proximal contact of class II composite restorations, according to the studies.

## Introduction and background

Composite resin has found extensive application as a direct restorative material due to its cost-effectiveness, enhanced visual appeal, and compatibility [1]. The American Dental Association (ADA) reports that approximately 69% of patients who seek dental treatment undergo direct restorative procedures. Among these procedures, 50% are classified as class II direct restorations, with composite resin restorations comprising 70% of the total restorations [2].

Direct class II composite restorations may present a more formidable challenge for the dentist in comparison to amalgam restorations, which can be compressed against the matrix, owing to their viscoelastic properties [1]. When restoring the proximal surfaces of posterior teeth, dentists frequently encounter the difficulty of reproducing the proximal contact and contour that are intrinsic to the unrestored teeth. This facilitates the preservation of a healthy interdental papilla and the prevention of periodontal disease and caries-causing food accumulation [3].

Proximal contact, also known as the contact area, refers to the area where the proximal surfaces of adjacent molars come into contact [4]. By transmitting occlusal forces along the long axis of teeth, interproximal contact achieves the arch's desired stability [3]. In order to guarantee the durability of restorations made from class II composite resin, it is imperative that both functional and aesthetic requirements are met. This encompasses the choice of a matrix system that replicates the optimal proximal contact subsequent to the insertion of restorations made of class II composite resin [4]. A dental matrix is a type of adhesive band that imitates the anatomical attributes of the tooth requiring restoration through surface adhesion. Matrix systems that are commercially accessible comprise linear or pre-curved sections, as well as non-circular or circumferential matrices. Additionally, there are metal or plastic matrix bands, which may or may not have separation rings [5]. Despite the widespread use of the sectional matrix in conjunction with class II composite resin restorations, its clinical application is still limited, primarily because of the matrix's rounded contour, which in some cases does not conform to the natural proximal contour of teeth. The close interdental contacts with the sectional matrix may also make it challenging to insert the matrix without inducing a dent [6].

Consequently, the objective of this systematic review is to assemble current research conducted on the various matrix systems utilized in class II composite restorations and assess their impact on the pre-existing composite proximal contact.

Materials and methods

Study Protocol and Registration

This study was registered at the research center of the College of Dentistry at Princess Nourah University with the registration number (2022/CDS/RD/07) and was approved by the Institutional Review Board (IRB) under the number (22-0471). This systematic review was carried out in accordance with the Preferred Reporting Items for Systematic Reviews Meta-Analysis (PRISMA) guidelines.

PICO Question and Eligibility Criteria

To facilitate an analysis of the extant literature and establish a clear path for the investigation, the subsequent research inquiry was formulated: Can close proximal contact be replicated through the utilization of various matrix systems (sectional matrix, circumferential) in class II direct composite restorations for adult posterior teeth?

The population/problem, intervention, comparison, and outcome of the study were established according to the PICO question.

P: Adult posteriors restored with Class II composite restorations.

I: Using different matrix systems.

C: Natural tooth anatomy.

O: Measuring proximal contact tightness.

Inclusion Criteria

The study included randomized clinical trials that assessed the matrix system employed in LCASS II composite restorations. The review exclusively considered articles that were published in the English language and contained their complete texts.

Exclusion Criteria

The study precluded all other types of research designs, including but not limited to theoretical reviews, retrospective studies, prospective studies, interventions, observational, in vitro, and clinical guides. All restorations were excluded, with the exception of composites. Research studies that were deemed inadequate by the Effective Public Health Practice Project (EPHPP) were excluded.

Search Strategy and Data Extraction

A comprehensive search was performed across the following five electronic databases: EMBASE, Web of Science, PubMed, Scopus, and Cochrane. This method is monitored by a panel of three unbiased evaluators. The preliminary phase entailed evaluating the title and abstract in order to ascertain potential articles. Following that, duplicate references were removed, and a thorough examination was conducted on the complete texts of the remaining articles. Inconsistencies were resolved by means of a consensus-building procedure involving all five reviewers.

Assessment of Methodological Quality

Three evaluators conducted a critical evaluation of the studies that were included in the analysis. A fourth reviewer served as a mediator in this process. The assessment was carried out utilizing the EPHPP instrument. The grading system employed in this evaluation method establishes a standard for the overall quality of a study; it categorizes the research as strong, moderate, or ineffectual.

Quality Assessment of the Studies

The quality of the included studies was evaluated using the EPHPP instrument, which determined that six of them were rated as moderate. 

## Review

In order to successfully restore a proximal surface, it is necessary to attain marginal adaptation, marginal ridge placement, and anatomically accurate contour and contact points [7]. The preservation of periodontal health and the integrity of the dental arch are significantly influenced by the adequacy of proximal contact and marginal adaptation [8]. By maintaining tight proximal contact and optimizing reconstruction of the proximal surface, it is possible to prevent food impaction, subsequent periodontal disease, and the formation of approximal carious lesions. This is achieved by taking into account both anatomical and occlusal factors, which serve to stabilize, align, and support the dentition while also protecting the interdental gingival papilla [7]. The purpose of this systematic review was to evaluate the efficacy of randomized controlled trials (RCTs) pertaining to proximal contact tightness and examine the diverse matrix systems utilized in Class II composite restorations. In comparison to the circumferential matrix system, the sectional matrix produced more secure proximal contact, which is the primary finding of this review. As reported by Loomans et al., the incorporation of the separation ring into the sectional matrix led to a greater degree of proximal tension in comparison to the circumferential matrix. Two varieties of sectional matrices were utilized in this randomized clinical trial: the Palodent matrix system and the contact matrix system. The circumferential matrices utilized were Tofflemire [9]. This result was also observed in three additional RCTs, which concluded that the sectional matrix with a separation ring provided the optimal contact point for proximal contact as opposed to circumferential [10-12]. The intensity of the proximal contact is suggested to be enhanced by combining a separation ring with the sectional matrix, according to all the studies included in this analysis. Nevertheless, there is currently no research that compares the effectiveness of utilizing a sectional matrix with or without a separating ring.

Additionally, a significant discovery of this research was the absence of any distinction between translucent and metallic matrix systems. Three RCTs are in agreement with these results [13-15]. The research conducted by Gomes et al. employed two distinct circumferential matrix systems: Unimatrix, a self-regulating polyester matrix, and Tofflemire, a metallic circumferential matrix [11]. The researchers reached the conclusion that no statistically significant distinction existed between the two matrices. Demarco et al. conducted an additional RCT utilizing a sectional matrix in place of the metallic matrix system, and their findings were comparable [14]. The type of matrix systems utilized in the RCT conducted by Prakki et al. was not specified; this is a limitation that must be addressed [13]. There was no notable distinction observed in terms of the quantity of surfaces incorporated (MO, DO) or the cavity's location (mesially or distally). However, the contact tightness exhibited a comparatively smaller increase in the three-surface cavities in contrast to the two-surface cavities [12].

An additional aspect that warrants consideration is the resin composite. Goncalves et al. compared the differences in proximal contact between two varieties of resin composites, Filtek P60 and Filtek P90 (is P60 hybrid?). The study's findings indicated that no statistically significant distinction existed among the categories [15]. In contrast, the approach taken by Prakki et al. in their other study, which centered on the method of composite placement, involved incremental insertion and prepolymerized particles of the identical resin composite. According to the findings of this investigation, no statistically significant difference was observed [12]. According to these results, the resin composite will have no effect on the tightness of proximal contact. Furthermore, the reliability of this finding is compromised as a result of the restricted range of resin composites utilized and the small number of RCTs that assessed proximal contact with multiple composite types.

Loomans et al. conducted a study that suggests that the proficiency of the operator performing the experimental procedure may exert a substantial influence on the restoration's effectiveness. The operators in this specific inquiry were undergraduate students; consequently, 739 occurrences of successful open contact were recorded, representing 61.6% of the overall sample size [16]. While the qualifications and level of expertise of the operators were not explicitly stated in the remaining studies, their scope was restricted to one or two operators. By employing this methodology, the accuracy and reliability of the provided restorations are improved, thereby augmenting their overall caliber.

In relation to the cavity's location, two research studies have reached the conclusion that there are no significant statistical differences in the quality of restorations or proximal tightness between the distal-occlusal and mesial-occlusal surfaces that are affected [17,18]. With regard to the quantity of surfaces, the two-surface cavity and the three-surface cavity lack any observable differentiation. On the contrary, previous research has shown that the increase in contact tension for three-surface cavities is significantly less than that of two-surface cavities [12].

A multitude of methodologies have been applied with the aim of objectively quantifying the proximity of contact. An evidence-based review of dental matrix systems that measure interproximal and interdental frictional forces using the following instruments: three-dimensional (3D) imaging, interdental metal strips, a tooth pressure meter, and a digital tension transducer. The tooth pressure meter (TPM) is regarded as an objective and precise instrument for determining the force of proximal contact. A substantial metallic strip is affixed to the TPM apparatus and occlusally inserted into the interdental space. Two randomized clinical trials utilized this device, whereas the remaining RCTs assessed participants using bite radiographs or dental floss, both of which are more subjective in nature than TPM.

Considerable attention must be devoted to the follow-up period in order to evaluate the quality of the restorations. Following-up periods were present in only four of the studies that were included, and the evaluation periods in the included RCTs varied from six months to four years. Loomans et al. determined, based on their investigation of long-term changes in proximal contact tightness, that immediately after treatment, proximal contact that is too tight tends to decrease, whereas contact that is too loose remains unchanged [17]. In contrast, two additional RCTs conducted by Prakki et al. and Goncalves et al. assessed the duration for a maximum of 18 months. The aforementioned investigations reached the conclusion that no alterations in proximal contact tightness were statistically significant [13,15]. Another RCT conducted a four-year evaluation. When metallic and translucent matrices were utilized, the quality of restorations as measured by clinical dimensions including marginal adaptation, marginal staining, and texture declined significantly, according to the findings of this study. Nevertheless, during the four-year evaluation period, a notable decline in restoration quality was identified solely in relation to translucent matrices. This decline was assessed in terms of proximal contacts, occlusal function, and color match [14].

According to the studies mentioned previously, the durability of restorations might decrease as time passes. However, when the form of matrix is considered, the quality disparity becomes insignificant.

Results

*Search Strategy* 

**Figure 1:**
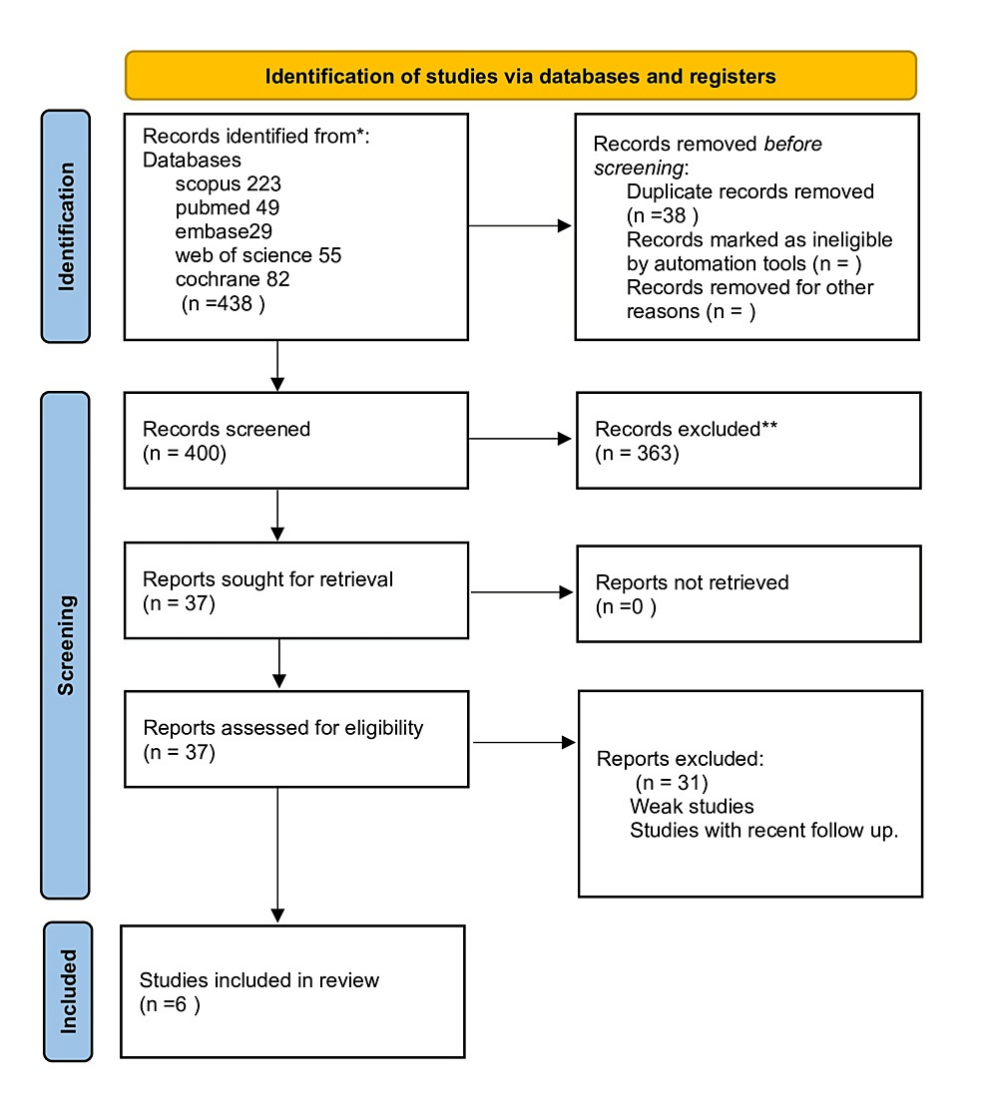
PRISMA 2020 flow diagram PRISMA: Preferred Reporting Items for Systematic Reviews Meta-Analysis

The electronic literature search yielded a cumulative sum of 223 studies from Scopus, 49 studies from PubMed, 29 studies from Embase, 55 studies from the Web of Science, and 82 studies from the Cochrane Library. The study selection procedure is depicted in Figure 1, which employs a PRISMA flowchart. Six investigations were, in conclusion, integrated into this review. 

Descriptive Analysis

The six studies incorporated in the systematic review and published from 2007 to 2018 are presented in Table 1. Aside from one clinical trial, all of the investigations were randomized controlled trials. Furthermore, the evaluation period consisted of either an extended follow-up or a baseline assessment.

**Table 1:** Study characteristics

Study	Dropouts	Evaluation period	Number of the operators	Study design	Dropouts	Methodological quality assessment using EPHPP (Effective Public Health Practice Project) global rate
Ahmad et al.,2018 [10]	Not applicable	Baseline evaluation	Multiple operators	RCT (Randomized Clinical Trials)	Not applicable	Moderate
Loomans et al.,2006 [9]	Not mentioned	Baseline, 6 months evaluation	Not Mentioned	RCT	Not mentioned	Moderate
Demarco et al., 2010 [14]	After 1 year: 86.2% After 2 years: 71.5% After 4 years: 65%	Baseline, one year, 2 years and 4 years Evaluation	One operator	RCT	After 1 year: 86.2% After 2 years: 71.5% After 4 years: 65%	Moderate
Wirsching et al., 2011 [12]	Not applicable	Baseline Evaluation	One operator	CCT (Controlled Clinical Trial)	Not applicable	Moderate
Gonçalves et al., 2012 [15]	4%	Baseline, 6 months evaluation	One operator	RCT	4%	Moderate
Gomes et al., 2015 [11]	Not applicable	Baseline evaluation	One operator	RCT	Not applicable	Moderate

The clinical technique, matrix system, type of composite resin, number of teeth, and surface restored are detailed in Table 2. Comparing circumferential and sectional matrix systems comprised half of the studies, whereas comparing polyester and metal matrix systems or composite varieties comprised the other half. The surface involved was mentioned in three studies pertaining to surface restoration. Certain studies neglected to specify the number of surfaces or teeth that were examined.

**Table 2:** Methodology

Study	Composite type	Matrix type	Surface	Sample size
Ahmad et al., 2018 [10]	hybrid‑filled composite.	Circumferential matrix system Sectional matrix system	Not mentioned	1200
Loomans et al., 2006 [9]	Hybrid composite with a high fill strength (Clearfil AP-X (A3), Kuraray Co., Osaka, Japan)	Tofflemire retainer (Produits Dentaire Palodent Matrix System (Dentsply Caulk) Contact Matrix System	Not mentioned	52
Demarco et al., 2010 [14]	Filtek P-60, 1LG/2ME/1KL, 2PE, 3M ESPE Hybrid-filled production number?	Metallic matrix and wooden wedge Polyester matrix and reflective wedge.	Not mentioned	109
Wirsching et al., 2011 [12]	Herculite, a hybrid-filled composite manufactured by Kerr Corporation in West Collins, USA.	Sectional matrix system combined with a separation ring (Palodent) Circumferential matrix system in combination with a retainer (Tofflemire).	MO/DO/MOD	85
Santos Gonçalves et al., 2012 [15]	Filtek P60, control group Filtek P90, test group	circumferential steel Matrix with a Tofflemire retainer and wooden wedges were used	MO DO MOD	50
Gomes et al., 2015 [11]	Charisma micro hybrid composite resin	Tofflemire Unimatrix sectioned metal matrix Unimatrix self-regulating polyester matrix	MO / DO	30

A comprehensive summary of the conclusions and findings obtained from the chosen studies is provided in Table 3, with an emphasis on the elucidation of proximal contact. The assessment of contact subsequent to the procedure was carried out in two randomized controlled trials employing the TPM as the designated instrument of measurement. The cumulative result is specific to each individual investigation. Furthermore, comprehensive documentation exists regarding the quantity and fundamental factors that contribute to the incidence of failure.

**Table 3:** Results

Study	Result / Conclusion	Cause of failure	Number of failures	Description of contact
Ahmad et al., 2018 [10]	Open contacts were identified in 739 (61.6%) of the teeth that were examined. Tight contacts were detected in 72 (6 percent) of the teeth. Optimal contacts were detected in 389 teeth, accounting for 32.4% of the total. All 389 (100%) optimal contact points were identified in teeth that underwent restoration utilizing the sectional matrix band system. A significant correlation was observed between open contact sites and the circumferential matrix band system.	Open contact	739 cases (61.6%)	Open contact Tight contact Optimum contact
Loomans et al.,2006 [9]	The implementation of separation rings and sectional matrices produced a more robust proximal contact in comparison to the circumferential matrix system.	Not mentioned	Not mentioned	Tight Proximal Contact Equal or looser proximal contact
Demarco et al., 2010 [14]	At the four-year evaluation, the clinical aspects of marginal adaptation, marginal staining, and roughness for metallic and translucent matrix restorations exhibited a statistically significant decline in quality (p<0.05). At the 4-year evaluation, only translucent matrices exhibited a statistically significant decline in restoration quality, as determined by occlusal and proximal contacts, color match, and proximal contacts (p<0.001).	Caries / Pulp necrosis / fracture	2 cases (1.8%) After 1 year 5 cases (4.6%) After 2 years 7 cases (6.4%) After 4 years	A Normal B Heavy C Light D Open >> no need
Wirsching et al., 2011 [12]	The utilization of the separation ring with two-surface cavities led to proximal contacts that were statistically substantially more snug at both the mesial and distal sites. compared to the use of the circumferential. Regarding the three-surface (MOD) cavities no statistically significant differences were found between the mesial and distal.	Not mentioned	Not mentioned	Proximal contact strength (tightness) was measured
Gonçalves et al., 2012 [15]	At baseline:94% of control was A 92% of test were A 6 months: 93.75% of control were A 85.42% of test were A	Not mentioned	1 case (2.08%) After 6 months	ALPHA Normal BRAVO Moderate CHARLIE Absent
Gomes et al., 2015 [11]	Proximal contact A total of seventeen (57%) of the thirty restorations received correct evaluations, with sectional matrix receiving the maximum frequency of correct assessments.	Under contour	9 cases (30%)	Correct Incorrect

Limitations

The principal limitation is the number of articles that are supported by evidence. Using the EPHPP assessment instrument, we evaluated the quality of the seven reviewed RCTs; all but one were of moderate quality, and the last one was deemed to be of poor quality. This underscores the necessity for robust RCTs grounded in evidence to examine the distinctions among matrix systems.

To ascertain the quality of restorations, it is imperative that their evaluation incorporate a follow-up period. However, of the reviewed RCTs, only four had a follow-up period, and neither the assessment methodologies nor the proximal contact for restorations were standardized. Determining changes in proximal contact tension in an accurate and objective manner necessitates RCTs with an evaluation period of at least six months.

## Conclusions

Based on the obtained results obtained from various studies, the subsequent conclusions were drawn. The efficiency of the proximal contour is influenced by the type of matrix system utilized. Specifically, the G2-SME yielded a greater proportion of accurate proximal anatomical contours in comparison to the G1-MMW and G3-PMR. There was no difference in the occurrence of incorrect proximal contacts based on the placement of the restoration (mesial or distal). Therefore, the utilization of sectional matrix systems significantly improves the proximal contacts of class II composite restorations in comparison to other matrix systems.
